# Hypoxic exosomes facilitate angiogenesis and metastasis in esophageal squamous cell carcinoma through altering the phenotype and transcriptome of endothelial cells

**DOI:** 10.1186/s13046-019-1384-8

**Published:** 2019-09-05

**Authors:** Yu Mao, Yimin Wang, Lixin Dong, Yunjie Zhang, Yanqiu Zhang, Chao Wang, Qiang Zhang, Sen Yang, Liyan Cao, Xinyuan Zhang, Xin Li, Zhanzhao Fu

**Affiliations:** 1 Department of Oncology, First Hospital of Qinhuangdao, Wenhua Road No. 258, Haigang District, Qinhuangdao, 066000 Hebei China; 2 Department of General Surgery, First Hospital of Qinhuangdao, Hebei Medical University, Qinhuangdao, China; 3 Department of Thoracic Surgery, First Hospital of Qinhuangdao, Qinhuangdao, China

**Keywords:** ESCC, Angiogensis, Exosomes, Metastasis

## Abstract

**Background:**

In cancer progression, hypoxia, or low oxygen tension, is a major regulator of tumor aggressiveness and metastasis. However, how cancer cells adapt to the hypoxia and communicate with other mesenchymal cells in microenvironment during tumor development remains to be elucidated. Here, we investigated the involvement of exosomes in modulating angiogenesis and enhancing metastasis in esophageal squamous cell carcinoma (ESCC).

**Methods:**

Differential centrifugation, transmission electron microscopy and nanoparticle tracking analysis were used to isolate and characterize exosomes. Colony formation and transwell assay were performed to assess the proliferation, migration and invasion of human umbilical vein endothelial cells (HUVECs). The tube formation assay and matrigel plug assay were used to evaluate the vascular formation ability of HUVECs in vitro and in vivo respectively. An in vivo nude mice model was established to detect the regulatory role of exosomes in ESCC progression. Microarray analysis was performed to analyze the transcriptome profiles in HUVECs.

**Results:**

Exosomes derived from ESCC cells cultured under hypoxia played a better role in promoting proliferation, migration, invasion and tube formation of HUVECs in vitro and in vivo than exosomes from ESCC cells cultured under normoxia. Moreover, hypoxic exosomes significantly enhanced the tumor growth and lung metastasis compared with normoxic exosomes in nude mice models. Interestingly, endothelial cells were programmed by hypoxic and normoxic exosomes from ESCC cells which altered the transcriptome profile of HUVECs.

**Conclusions:**

Taken together, our data identified an angiogenic role of exosomes from ESCC cells which shed light on the further application of exosomes as valuable therapeutic target for ESCC.

**Electronic supplementary material:**

The online version of this article (10.1186/s13046-019-1384-8) contains supplementary material, which is available to authorized users.

## Background

Esophageal cancer is one of the most common malignant tumors worldwide which seriously threatens human health [1]. Esophageal squamous cell carcinoma (ESCC) is the predominant form of esophageal cancer worldwide [2]. Conventional therapeutic strategies such as radiotherapy, chemotherapy and surgery show limited effect for ESCC treatment, especially for advanced ESCC with metastasis [3]. During cancer progression, tumors cannot grow beyond 1–2 mm without a vascular supply, due to an insufficient supply of oxygen, hypoxia, appears [4, 5]. Hypoxia is a major driver of tumor angiogenesis [5, 6]. As a multi-step physiological process, angiogenesis comprises several sequential steps involving the proliferation, migration, and morphogenesis of endothelial cells [4, 7, 8] . In tumor microenvironment (TEM), such coordination is partially accomplished by the transfer of angiogenic factors from tumor cells to endothelial cells [4, 9].

Recent studies has verified the potential role of exosomes as important signaling entities in the cross-talk between various cell types. Exosomes are vesicles of endocytic origin released by many cells [10, 11]. The involvement of exosomes in esophageal cancer proliferation, metastasis, and drug resistance is becoming increasingly apparent [12–18]. Besides, exosomes can reflect the hypoxic status of cancer cells and further help them adapt to hypoxia through communicating with their surrounding microenvironment during tumor development [19]. However, whether exosomes play a role in modulating angiogenesis and, hence, helping the ESCC cells conquer the hypoxic microenvironment remains to be elucidated.

In the present study, the potential effect of exosomes from ESCC on endothelial cells in TEM was firstly explored. Moreover, by culturing the ESCC in hypoxic condition, we demonstrated that hypoxia might alter the content of exosomes secreted by ESCC cells and further modulate the activation of endothelial cells which internalized these exosomes.

## Material and methods

### Cell lines and cell culture

In order to obtain the exosomes-depleted fetal bovine serum (FBS), FBS was firstly centrifuged at 100,000×g for 12 h in an ultracentrifuge (Beckman Coulter, Optima L-100XP, USA) at 4 °C and then discarded the pellet.

ECA109, KYSE410 and HET-1A cell lines were purchased from American Type Culture Collection. HET-1A cell line was cultured in Epithelial Cell Growth Basal Medium (Lonza, Switzerland). ECA109 and KYSE410 cell lines were cultured in RPMI 1640 (Life Technologies/Invitrogen) supplemented with 10% FBS (Gibco, New Zealand) and 1% penicillin/streptomycin (Gibco, New Zealand).

Primary human umbilical vein endothelial cells (HUVECs) were also purchased from American Type Culture Collection and maintained in endothelial cell medium (ECM) (Science cell, USA). All cells were cultivated in a humidified incubator at 37 °C with 5% CO_2_. For hypoxia experiments, cells were incubated in a humidified 5% CO_2_ and 0.5% O_2_ for 5 days at 37 °C.

### Exosomes isolation

Exosomes were extracted from ESCC cell culture medium using differential centrifugation. To this end, culture medium (9 ml) were collected from ESCC cells (1 × 10^7^) cultured at normoxic or hypoxic conditions in a 10 cm diameter petri dish. Culture medium were centrifuged at 300 g - 5 min to eliminate cell debris. Supernatant were further centrifuged at 16,500 g-30 min and 100,000 g-2 h. Finally, the exosome pellets were washed once in a large volume of phosphate buffer saline (PBS) and then followed by centrifugation (100,000 g-2 h). The exosomes were quantified by measuring the exosomal protein with BCA™ Protein Assay Kit (Pierce, USA).

### Transmission electron microscopy

The exosome-enriched suspension were suspended in 50 μL of PBS, fixed with paraformaldehyde and glutaraldehyde. Exosome sample was adsorbed onto a carbon-coated copper grid and immersed in phosphotungstic acid solution for 30 s. Then the samples were observed in a Zeiss transmission electron microscope (Zeiss, Germany).

### Nanoparticle tracking analysis

The number of exosomes and the size distribution were analyzed using the Nanosight (Malvern, UK) and NTA analytical software (version 2.3, Nanosight).

### Western blotting analysis

The purified exosomes and ECA109 or KYSE410 cells were lysed using RIPA buffer (Roche). The protein concentration of lysates was quantified with BCA™ Protein Assay Kit (Pierce, USA). Then proteins were separated by SDS-PAGE and transferred onto a PVDF membrane. The PVDF membrane was blocked with 5% non-fat milk and incubated with the primary antibodies (CD9 and TSG101) (Cell Signaling Technology, USA) overnight at 4 °C. The bands were probed with secondary antibody (Icllab, USA) and visualized by chemiluminescence (Millipore, MA, USA). The intensity of the protein bands was quantified by densitometry using Image J Software (National Institutes of Health). Each assay was repeated at least three times. One representative of three independent experiments was shown.

### Colony formation assay

As we previously depicted, HUVECs were seeded into 6-well plates (500 cells/well) and incubated with medium containing exosomes (25 μg /mL) or not for 2 weeks. The colonies were stained with crystal violet for 15 min and then counted [20, 21]. Each assay was repeated at least three times. One representative of three independent experiments was shown.

### Cell cycle analysis

As we have previously depicted, HUVECs were fixed with ice-cold ethanol for 24 h and then dyed with propidium iodide/RNase buffer (BD Biosciences, USA) for 30 min in a dark place. Samples were analyzed by BD FACS Calibur flow cytometer. Data was analyzed using Modfit [20]. Each assay was repeated at least three times. One representative of three independent experiments was shown.

### Invasion and migration assay

As we previously depicted, 0.1 mL FBS-free ECM containing 2 × 10^4^ HUVECs, in the presence of exosomes (25 μg /mL) or not, were seeded into the upper chamber of transwell chambers (24 wells) (Merck, Germany) coated with or without Matrigel (BD Biosciences, USA). 0.6 mL of the ECM containing 10% FBS was placed into the lower chamber. 48 h later, the bottom surface of the membranes were stained with crystal violet and photographed. The number of invasive cells were counted in three random microscopic fields under a light microscope [22] . Each assay was repeated at least three times. One representative of three independent experiments was shown.

### Exosomes labeling, internalization and confocal microscopy

Exosomes were labeled with PKH26 Red Fluorescent marker (PKH26GL, Sigma-Aldrich, Germany) as recommended by the manufacturer. HUVECs were incubated with labeled exosomes (25 μg /mL) for varying times. Then HUVECs were fixed and stained with Phalloidin-iFluor 488 Reagent (Abcam, UK) and DAPI (Solarbio, USA) according to the manufacturer’s instruction. The images were acquired with a Zeiss Laser Scanning Confocal Microscope (Zeiss, Germany).

### Matrigel tube formation assay

HUVECs were seeded into a Matrigel Basement Membrane Matrix (BD, New Jersey, USA) precoated 96-well plate at 2 × 10^4^ cells per well and cultured in ECM, in the presence of exosomes (25 μg /mL) or not. Calcein-AM (Sigma-Aldrich, Germany) was used to stain the HUVECs after seeding for 8 h. The tube-like structures were imaged using the Zeiss fluorescence inverted microscope (Zeiss, Germany). The angiogenic property was assessed by measuring the total branching length from three random microscopic fields using Image J software (National Institutes of Health). Each assay was repeated at least three times. One representative of three independent experiments was shown.

### In vivo matrigel plug assay

Five hundred ul of Matrigel (BD Biosciences) mixed with (25 μg /mL) normoxic or hypoxic exosomes was injected subcutaneously into the ventral region of BALB/c nude mice (Institute of Laboratory Animal Sciences, Chinese Academy of Medical Sciences (CAMS)). After inoculation for 7 days, the matrigel was excised and then fixed with formalin overnight, embedded in paraffin, and sectioned into slides. The plugs were stained with hematoxylin and eosin (H&E) and visualized using the Zeiss inverted microscope (Zeiss, Germany) [23]. Three mice were used for each group. One representative of three independent experiments was shown.

### Expression profile analysis of RNAs in exosomes

HUVECs were divided into three groups: HUVECs incubated without exosomes (control group), HUVECs incubated with exosomes (25 μg /mL) from KYSE410 which cultured in hypoxic environment (hypo-Exo group) and HUVECs incubated with exosomes (25 μg /mL) from KYSE410 which cultured in normoxic environment (norm-Exo group). After incubation for 12 h, the whole RNA of HUVECs was extracted using trizol (Thermo, USA). Gene expression profiling was performed by Beijing CNKINGBIO Biotechnology Company Limited using Clariom™ D Pico Assay, human (Affymetrix, USA) according to user guide.

Quantile normalization and subsequent data processing were performed using Applied Biosystems™ Transcriptome Analysis Console (TAC) Software (Affymetrix, USA). Heat maps representing differentially regulated genes were generated using R software (Vienna University of Economics and Business, Austria). According to the results of microarray, RNAs with fold change > 1.5 were marked as significantly differentially expressed genes.

### Gene annotation and pathway enrichment analysis

Differentially regulated mRNAs were put into gene ontology (GO) biological process enrichment and Kyoto Encyclopedia of Genes and Genomes (KEGG) signaling pathways analysis as previously depicted [20, 21, 24]. The results of bioinformatics analysis were plotted as bubble chart using R packaging ‘ggplot2’ [25, 26]. The interaction relationship between these mRNAs were evaluated by applying Search Tool for the Retrieval of Interacting Genes (STRING) which is an online tool designed to evaluate the protein–protein interaction (PPI) information [27]. Node connectivity (degree) were calculated using contextual hub analysis tool in Cytoscape software to identify hub genes [28, 29]. Nodes with degree > 10 were considered as highly connected nodes (hub genes).

### In vivo nude mice xenograft model

As we previously depicted, 2 × 10^6^ ECA109 or KYSE410 cells were inoculated subcutaneously to the hind limb of BALB/c nude mice (CAMS) to construct the ESCC xenograft model. Animals injected with ECA109 or KYSE410 cells were randomly divided into five groups. When the nude mice generate tumors with a size of 100 mm^3^, PBS, 10 μg exosomes from normal squamous esophageal epithelial cell line (HET-1A), exosome release inhibitor (GW4869, 1 mg/kg), 10 μg exosomes from normoxic ECA109 or KYSE410 cells (norm-Exo), or 10 μg exosomes from hypoxic ECA109 or KYSE410 cells (hypo-Exo) were then injected into the center of tumor sites every 2 days. The tumor size was measured every 5 days. After 40 days, the nude mice were sacrificed and tumors were taken out to measure the weight. The proliferation and angiogenesis status of tumor tissues and lung metastasis were determined using histological examination. Tumor volume was calculated with the formula: length × width^2^ × 0.5 [22, 30]. Three mice were used for each group. One representative of three independent experiments was shown.

### Immunohistochemistry

Primary tumor tissues and lungs were firstly fixed in formalin and embedded in paraffin, and then cutted into sections. The lung sections were then stained by hematoxylin and eosin. For immunohistochemistry staining, sections were incubated with primary antibodies (Ki-67, CD31) (Servicbio, China) at 4 °C overnight, followed by secondary antibody. The stained tumor sections were visualized using the Zeiss inverted microscope (Zeiss, Germany). The number of positive staining tumor cells were counted in three random microscopic fields.

### Statistical analysis

Statistical analysis was conducted using GraphPad Prism 6 software. Unpaired t-test was used to analyze the differences between two groups. Comparisons among more than two groups were performed using one-way ANOVA followed by Holm-Sidak’s multiple comparison tests. *P* value < 0.05 was considered significant. All data are expressed as mean ± standard deviation (SD).

## Results

### Characterization of exosomes from ESCC cells

**Fig. 1:**
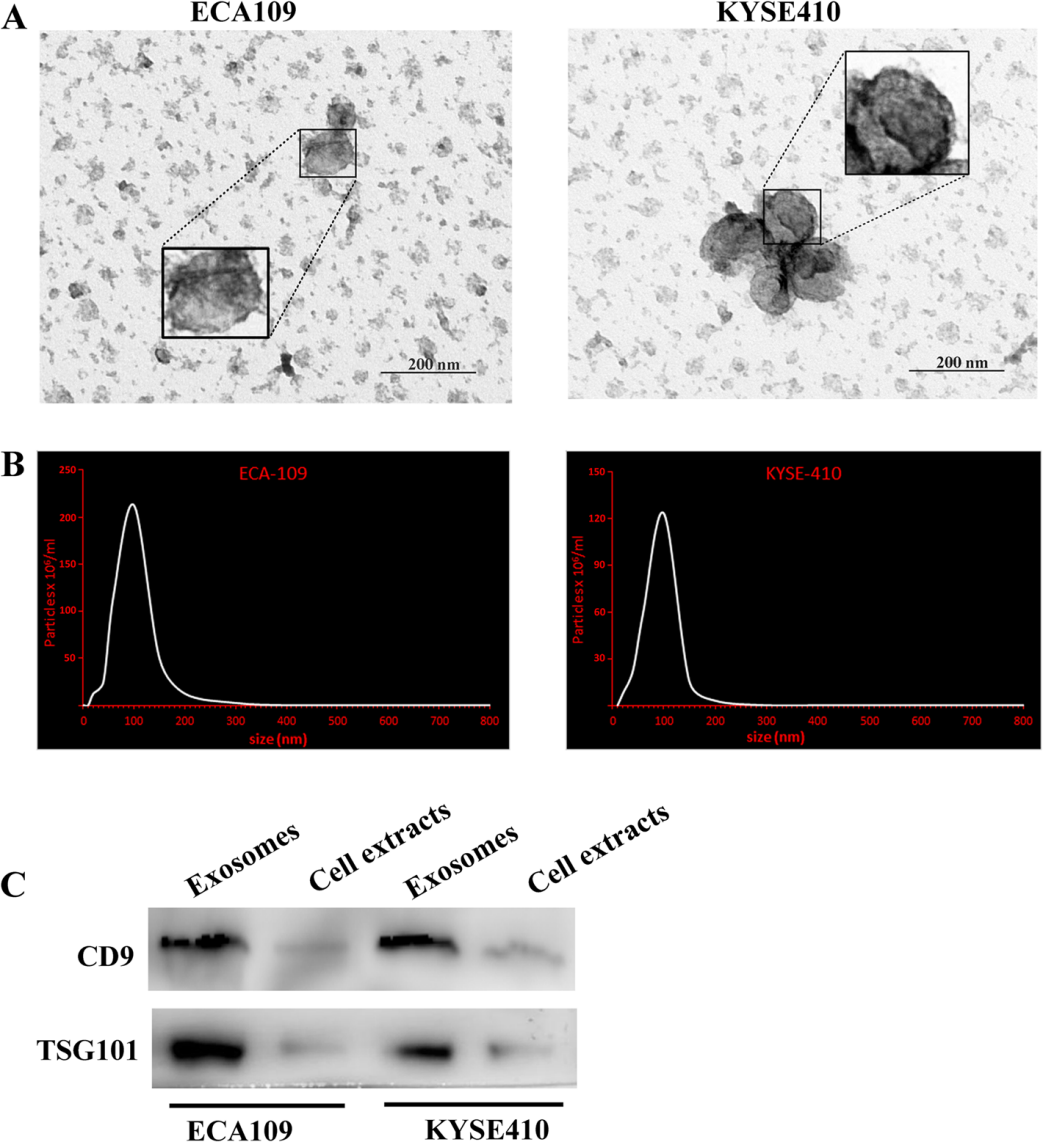
Identification of the purified extracellular vesicles. **a** Transmission electron micrographs of extracellular vesicles derived from ECA109 and KYSE410. **b** The nanoparticle concentration and size distribution of the extracellular vesicles derived from ECA109 and KYSE410. **c** The expression level of CD9 and TSG101 (exosome specific markers) in extracellular vesicles

The morphology of purified extracellular vesicles from the supernatant of ESCC cells (ECA109, KYSE410) cultured under normoxic conditions was visualized by transmission electron microscopy (Fig. 1a). NTA showed that the particle size distribution of purified extracellular vesicles were between 20 and 200 nm (Fig. 1b).

Western blotting analysis demonstrated that specific exosome markers (CD9 and TSG101) were enriched in purified extracellular vesicles from the supernatant of both ECA109 and KYSE410 (Fig. 1c). All together, these results confirmed that exosomes were extracted from the ESCC cell supernatant.

### ESCC cells derived exosomes were internalized by endothelial cells

**Fig. 2:**
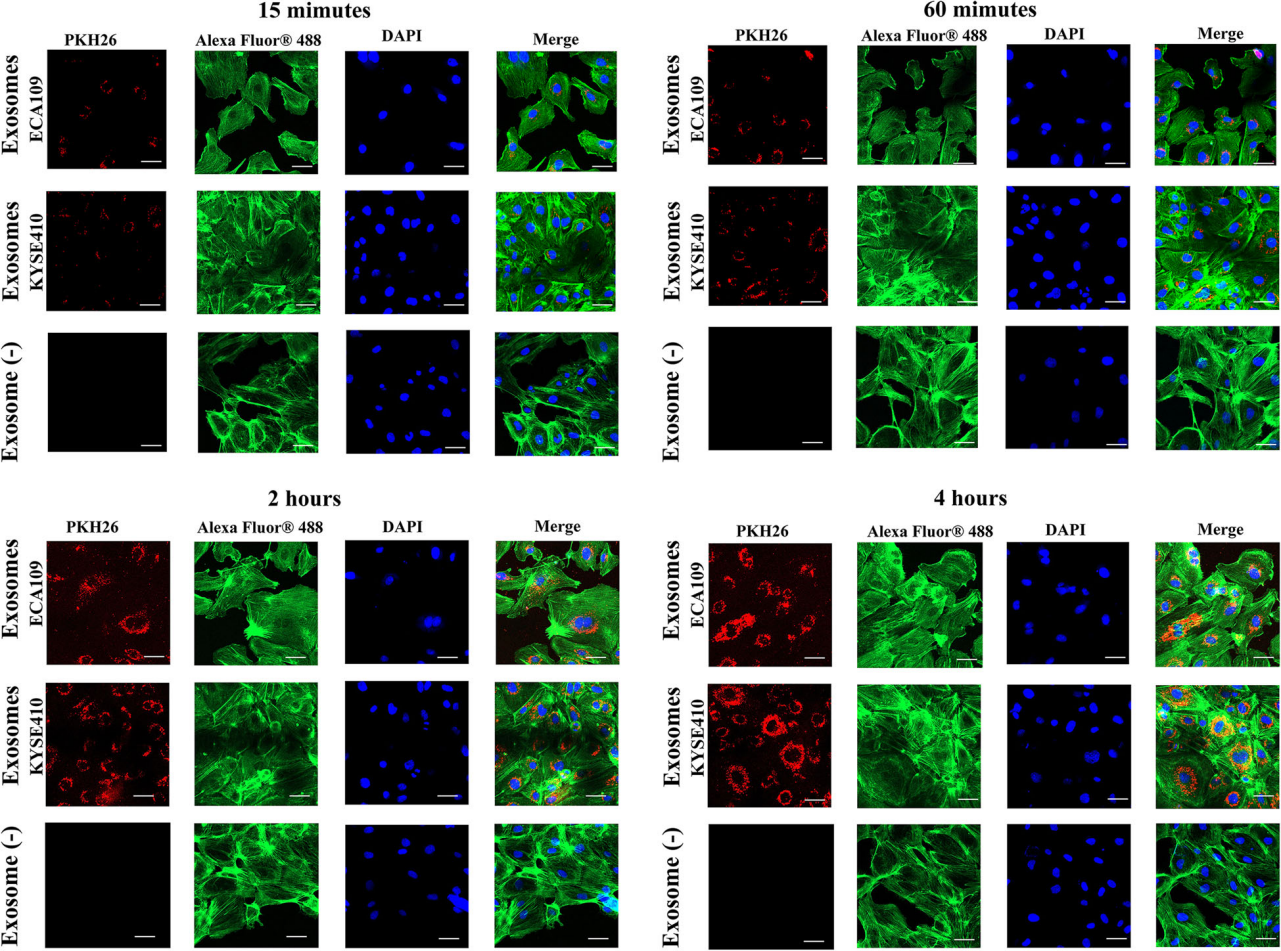
Uptake of exosomes derived from ECA109 and KYSE410 by HUVECs at 15 min, 60 min, 2 h and 4 h. HUVECs were cultured with exosomes (25 μg /mL) from ECA109, or exosomes (25 μg /mL) from KYSE410, or in the absence of exosomes (Exosome (−)). Fluorescence microscopy images showing the internalization of exosomes by HUVECs. Blue: Nucleus stained with DAPI. Red: PKH26-labeled exosomes. Green: Phalloidin-iFluor 488 Reagent. Scale bar, 50 μm

To investigate interactions between exosomes from ESCC cells and HUVECs, exosomes stained with the PKH26 were incubated with HUVECs. The uptake of exosomes in HUVECs was recorded by confocal microscopy at 15 min, 60 min, 2 h and 4 h. Then HUVECs were stained with iFluor 488 for Phalloidin and DAPI for nucleus. Figure 2 demonstrated that exosomes uptake by HUVECs started after 15 min of incubation and increased constantly over time. The internalized exosomes mainly located in the cytoplasm of endothelial cells. In contrast, no labeling was observed in the control group (HUVECs cultured without exosomes) (Exosome (−)). Our results here suggested the internalization of exosomes, from both ECA109 and KYSE410, by HUVECs.

### Hypoxic exosomes promoted endothelial cell proliferation, cell cycle progression and migration

**Fig. 3:**
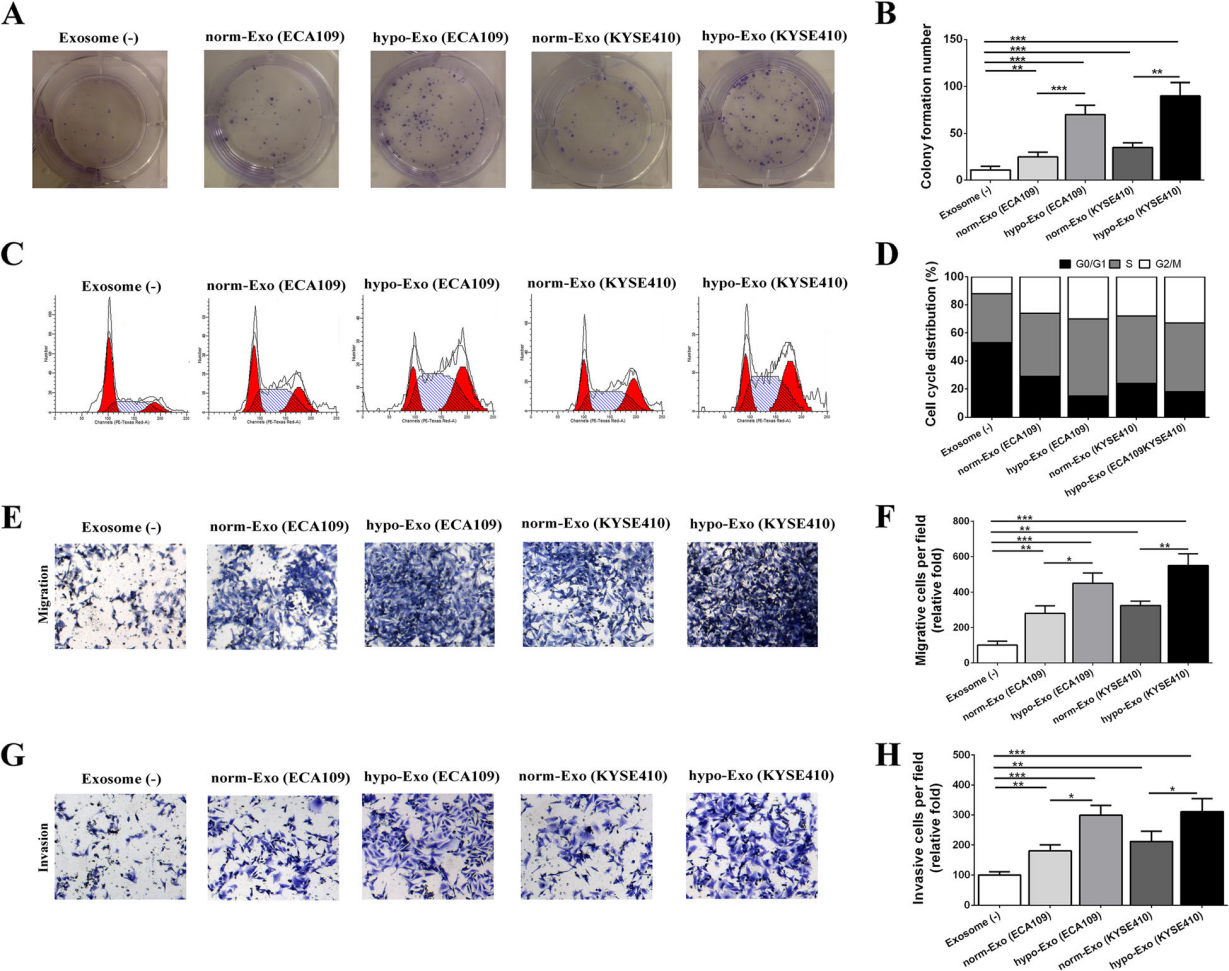
The regulatory role of normoxic and hypoxic exosomes in the proliferation, cell cycle distribution, migration and invasion of HUVECs. HUVECs were cultured with exosomes (25 μg /mL) from ECA109 that cultured in normoxic environment (norm-Exo (ECA109)) or hypoxic environment (hypo-Exo (ECA109)), or exosomes (25 μg /mL) from KYSE410 that cultured in normoxic environment (norm-Exo (KYSE410)) or hypoxic environment (hypo-Exo (KYSE410)), or in the absence of exosomes (Exosome (−)). The proliferation of HUVECs was detected by colony formation assay (**a**). The graph summarizes the results of three independent experiments (**b**). The cell cycle of HUVECs were analyzed by flow cytometry. Representative pictures of the cell cycle distributions in HUVECs (**c**). The graph summarizes the results of three independent experiments (**d**). Transwell assays were used to investigate the migratory (**e**) and invasive (**g**) abilities of HUVECs. The graph summarizes the results of three independent experiments of migration (**f**) and invasion assay (**h**). Data was presented as mean ± standard deviation (SD).*P < 0.05, **P < 0.01, ***P < 0.001

Exosomes were isolated from ESCC cells which cultured in hypoxic and normoxic environment respectively. Then we investigated the effects of normoxic exosomes (norm-Exo) and hypoxic exosomes (hypo-Exo) on HUVECs proliferation, migration and invasion. In the proliferation analysis, norm-Exo increased the colony formation number of HUVECs. Moreover, hypo-Exo played a better role in boosting the colony formation number than norm-Exo or control group (Fig. 3a-b). Follow-up cell cycle analysis demonstrated that norm-Exo promoted HUVECs proliferation by facilitating the cell cycle progression, leading to an increased S and G2/M population. Besides, hypo-Exo played a better role in facilitating the cell cycle progression in HUVECs. (Figure 3c-d). In transwell assay, norm-Exo enhanced the migration and invasion of HUVECs. Similar to the results of cell cycle, HUVECs cultured with hypo-Exo exhibited higher motility than those cultured with norm-Exo (Fig. 3e-h). These results suggest that hypoxic ESCC cell-derived exosomes promoted endothelial cell proliferation, migration and invasion in vitro.

### Hypoxic exosomes accelerated vascular formation in vitro and angiogenesis in vivo

**Fig. 4:**
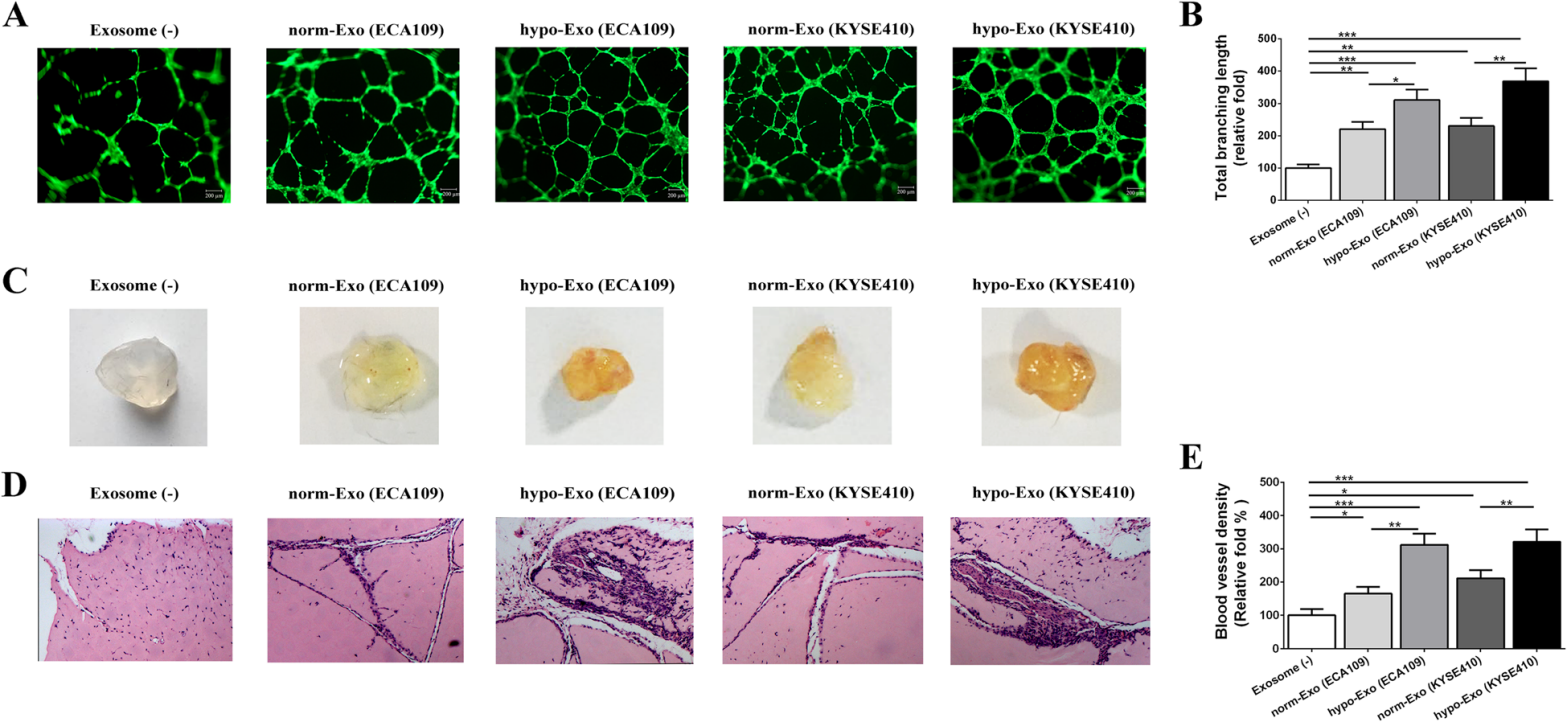
Hypoxic exosomes promoted angiogenesis in vitro and increased the vessel density in vivo. HUVECs were plated with matrigel and cultured with exosomes (25 μg /mL) or not. Representative pictures of tube formation were taken after stained with Calcein-AM (**a**). The tube formation ability was quantified by measuring the total branching length (**b**). Matrigel containing exosomes, or not, were injected subcutaneously into the nude mice. Representative images of the general observation of matrigel plugs (**c**). In vivo neovascularization induced by exosomes was measured by H&E staining. Representative pictures of neovascularization were shown in (**d**) and quantified for blood vessel density (**e**). Data was presented as mean ± standard deviation (SD). *P < 0.05, **P < 0.01, ***P < 0.001

In order to determine the effects of ESCC cell-derived exosomes on vascular formation ability of HUVECs, we performed the tube formation assay based on matrigel matrix. Firstly, we observed that both norm-Exo and hypo-Exo significantly increased the number of nodes and total tube length in network structures compared with those cultured without exosomes (Exosome (−)), indicating the pro-angiogenesis ability of exosomes from ESCC cells in vitro. Further analysis revealed that HUVECs cultured with hypo-Exo exhibited better vascular formation ability compared with those cultured with norm-Exo (Fig. 4a-b).

The angiogenic potential of exosomes from ESCC cells was further evaluated in vivo by examining the recruitment of vasculature into subcutaneously implanted Matrigel plugs containing exosomes. Figure 4c showed that the plugs containing norm-Exo or hypo-Exo became more vascularized than implants without exosomes (Exosome (−)).

Histological examination showed that matrigel plug containing norm-Exo or hypo-Exo showed much more micro-vessels. The vascular density of Matrigel plugs containing hypo-Exo were significantly higher than those containing norm-Exo. (Figure 4d-e) These data suggested that hypo-Exo played a better role for endothelial cells recruitment and vascular organization than norm-Exo both in vitro and in vivo.

### Hypoxic exosomes facilitated ESCC tumor growth, enhanced angiogenesis and promoted metastasis

**Fig. 5:**
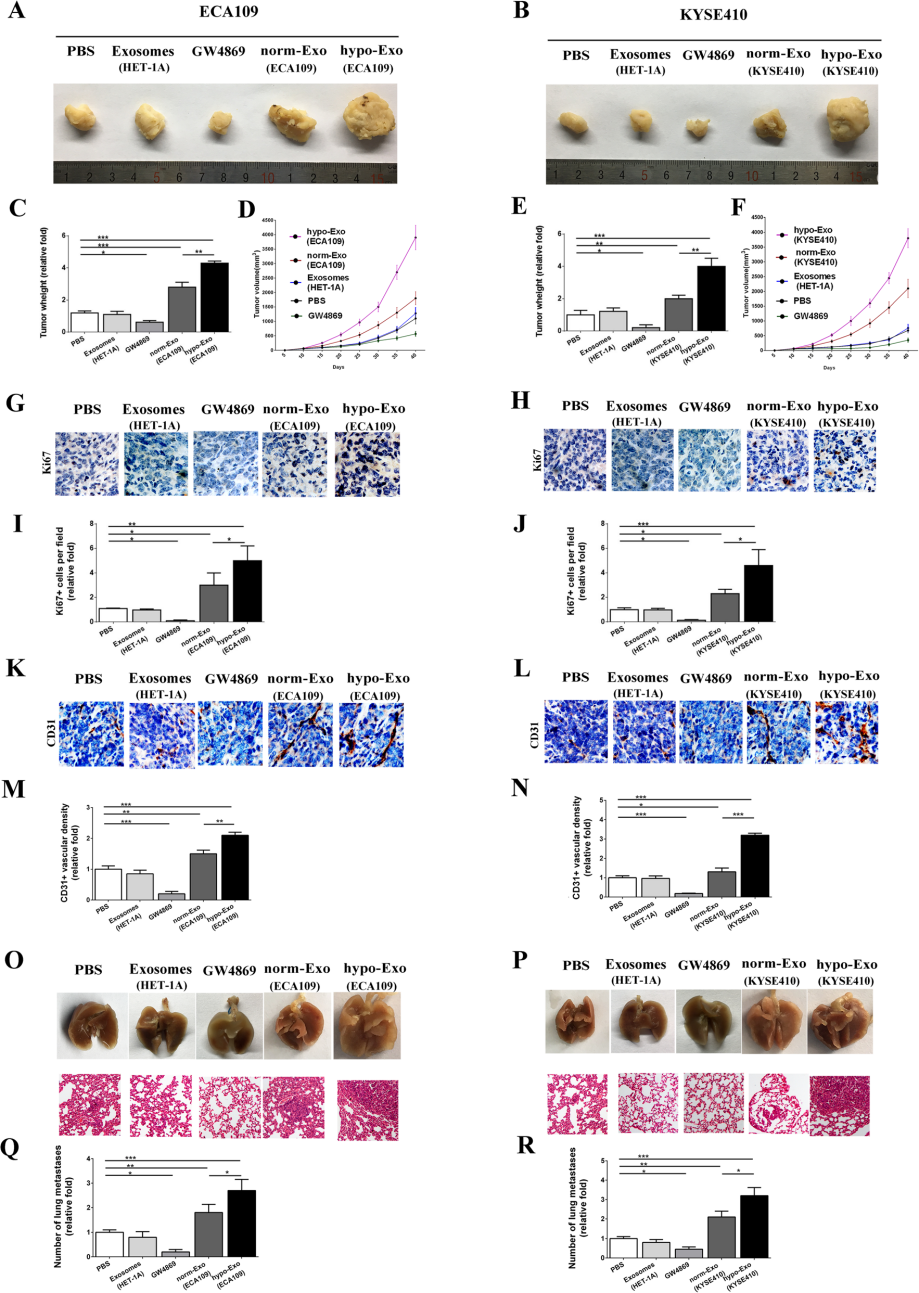
Hypoxic exosomes promoted tumor proliferation and angiogenesis in situ and facilitated lung metastasis. Xenograft transplanted tumor models were established through subcutaneously injecting ECA109 (**a**) or KYSE410 (**b**) into the nude mice. Then animals were randomly divided into five groups and injected with PBS, 10 μg exosomes from normal squamous esophageal epithelial cell line (HET-1A), exosome release inhibitor (GW4869, 1 mg/kg), 10 μg exosomes from normoxic ECA109 or KYSE410 cells (norm-Exo), or 10 μg exosomes from hypoxic ECA109 or KYSE410 cells (hypo-Exo) every 2 days (**a** and **b**). ECA109 or KYSE410 tumor weigh was measured and shown in **c** and **e**. Tumor growth curves for ECA109 or KYSE410 tumor models were shown in **d** and **f**. Then tumors were analyzed by immunofluorescence microscopy for Ki67 and CD31. Representative pictures of Ki67 were shown in **g** and **h**, and quantified for cell proliferation (**i** and **j**). Representative pictures of CD31 were shown in **k** and **l**, and quantified for vascular density (**m** and **n**). Representative images of the general observation the lungs with metastasis nodules and the corresponding H&E images of the tumor edges in lungs (**o** and **p**). The metastasis lung nodules were quantified (**q** and **r**). Data was presented as mean ± standard deviation (SD). *P < 0.05, **P < 0.01, ***P < 0.001

To further determine the angiogenic role of exosomes in vivo, an ESCC tumor-bearing nude mice model was established and treated with exosomes from HET-1A, exosome release inhibitor (GW4869), normoxic ESCC-secreted exosomes (norm-Exo) or hypoxic ESCC-secreted exosomes (hypo-Exo). In mice xenograft model, norm-Exo injection promoted the tumor proliferation and resulted in an increased tumor size. Moreover, administration of hypo-Exo substantially accelerated tumor expansion at later time points, resulting in a significant increase in final tumor volume and tumor weight compared with norm-Exo and control group. (Fig. 5a-f). In support of these data, tumors with hypo-Exo showed substantially enhanced tumor cell proliferation compared with norm-Exo and untreated controls with more tumor cells positively stained with Ki67 (Fig. 5g-j). CD31 immunohistochemical analysis suggested that ESCC tumors grown in the presence of hypo-Exo exhibited enhanced vascularization and significantly greater microvessel density, compared with tumors in norm-Exo and control group (Fig. 5k-n). Hence, although norm-Exo showed a tendency to stimulate tumor growth, hypo-Exo exosomes were significantly more potent.

Also, a significant larger number of lung metastasis nodules was observed in hypo-Exo group than norm-Exo and control group. Immunohistochemistry stain of the lungs showed that the metastasis nodules in hypo-Exo group were more extensive which was consistent with the general observation results (Fig. 5o-r).

Besides, exosomes from normal squamous esophageal epithelial cell line (HET-1A) had no significant effects on ESCC progression. However, exosome release inhibitor, GW4869, suppressed ESCC tumor proliferation or angiogenesis in situ or distant lung metastasis notably in both ECA109 and KYSE410 mice xenograft model.

### Microarray analysis of transcriptome in HUVECs treated with hypoxic and normoxic exosomes

**Fig. 6:**
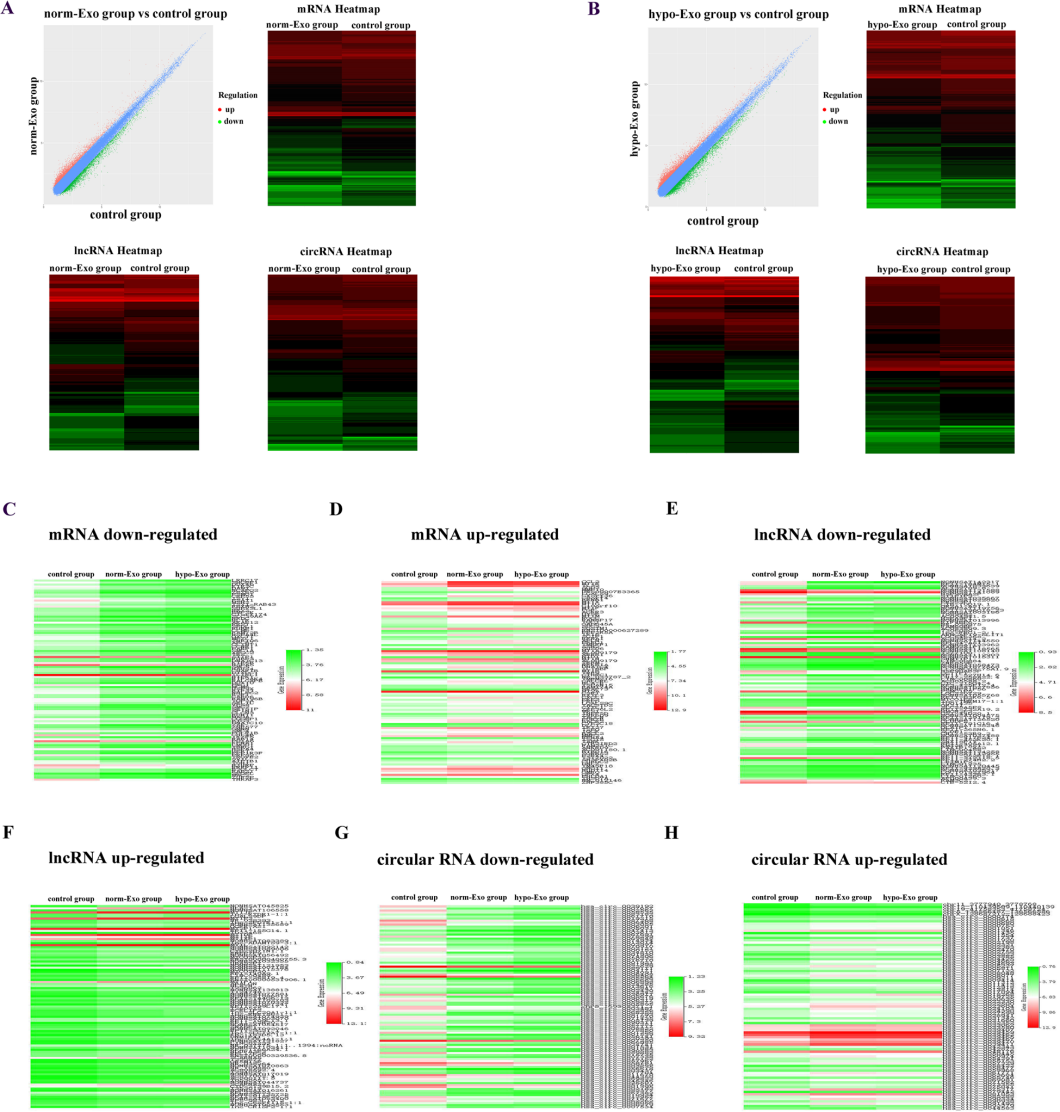
Microarray analysis revealed differentially expressed RNAs between different groups. **a** Scatter-Plot of differentially expressed RNAs variations between HUVECs in the control group and norm-Exo group. Dots above the top line (red) and below the bottom line (green) indicated the fold change of the RNAs is more than 1.5 between the two groups. Heat map of the dysregulated mRNA, lncRNA and circular RNA expression in control group and norm-Exo group. **b** Scatter-Plot of differentially expressed RNAs variations between HUVECs in the control group and hypo-Exo group. Heat map of the dysregulated mRNA, lncRNA and circular RNA expression in control group and hypo-Exo group. Eight hundred and thirty nine down-regulated mRNAs (**c**), 113 up-regulated mRNAs (**d**), 232 down-regulated lncRNAs (**e**), 99 up-regulated lncRNAs (**f**), 692 down-regulated circular RNAs (**g**) and 86 up-regulated circular RNAs (**h**) were identified according to the intersection of transcriptome between norm-Exo group and hypo-Exo group

To elucidate the functional mechanism and angiogenic effects of ESCC exosomes in endothelial cells, we compared the transcriptome in HUVECs which uptake the hypo-Exo or norm-Exo using microarray analysis. The experiment composed of three groups: HUVECs incubated without exosomes (control group), HUVECs incubated with exosomes form KYSE410 that cultured in normoxic condition (norm-Exo group) and HUVECs incubated with exosomes from KYSE410 that cultured in hypoxic condition (hypo-Exo group). By comparing the transcriptome of HUVECs in norm-Exo and control group, a total of 1656 mRNAs, 1002 lncRNAs and 1312 circular RNAs were dysregulated in HUVECs after the internalization of normoxic ESCC exosomes (Fig. 6a). On the other side, a total of 2171 mRNAs, 1533 lncRNAs and 1698 circular RNAs were dysregulated in HUVECs after the internalization of hypoxic ESCC exosomes (Fig. 6b) (Additional files 1 and 2).

Results aforementioned showed that hypoxia facilitate the angiogenic effects of exosomes, hence, we focused on the RNAs which experienced an up-or down-regulation in both norm-Exo group and hypo-Exo group. According to the intersection of transcriptome, we identified 839 mRNAs which down-regulated in both norm-Exo and hypo-Exp groups (Fig. 6c). In this way, 113 up-regulated mRNAs (Fig. 6d), 232 down-regulated lncRNAs (Fig. 6e), 99 up-regulated lncRNAs (Fig. 6f), 692 down-regulated circular RNAs (Fig. 6g) and 86 up-regulated circular RNAs (Fig. 6h) were identified in both norm-Exo and hypo-Exp groups.

### Function annotation and signaling pathways analysis of differentially expressed genes

**Fig. 7:**
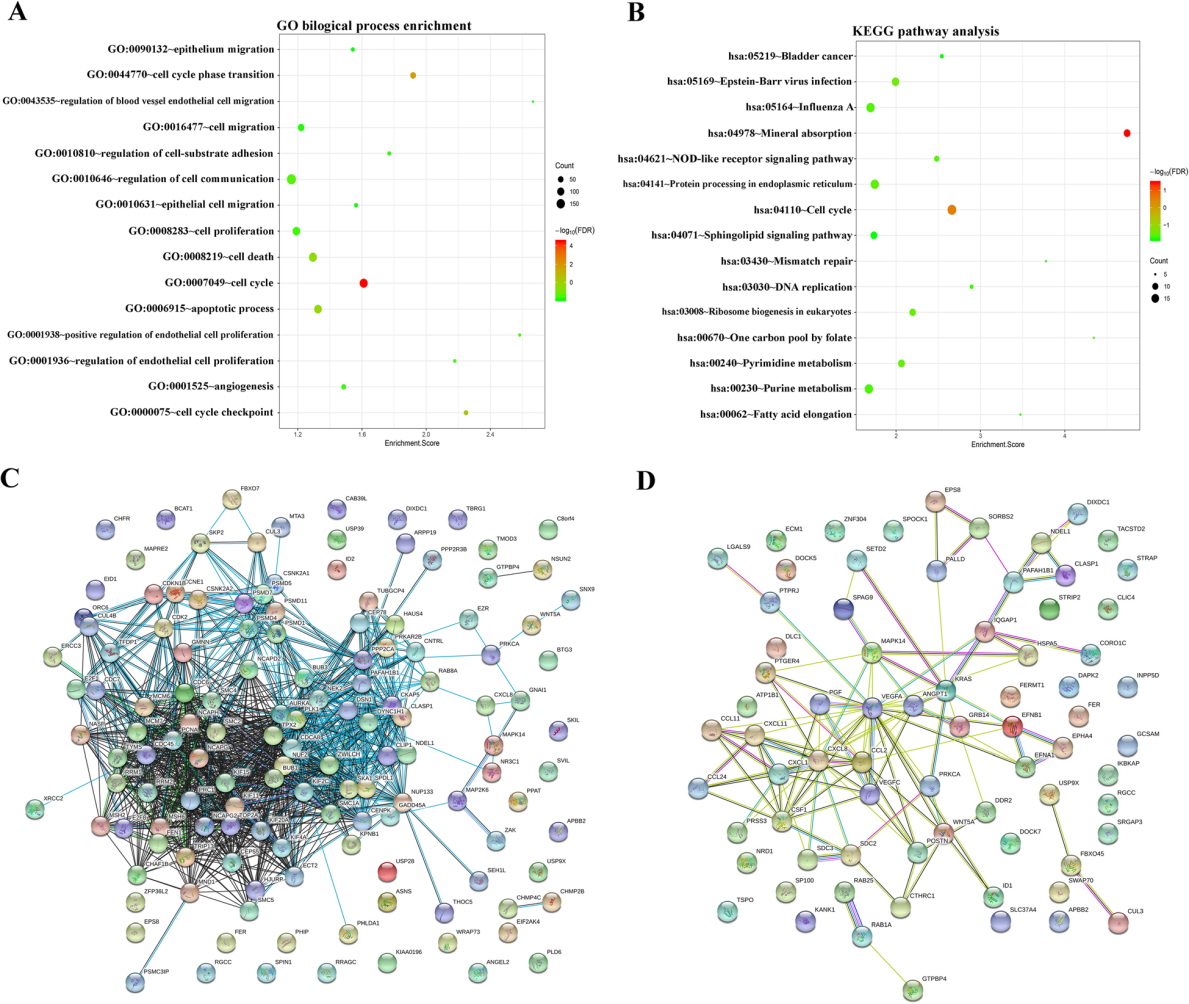
Function annotation of dysregulated genes and hub genes identification. The results of GO biological process enrichment (**a**) and KEGG signaling pathways analysis (**b**) were presented as bubble chart. The size of bubble indicate the number of genes enriched in corresponding annotation and the color indicate the –log10 value of false discovery rate (FDR). The PPI network for dysregulated genes that enrichment in cell cycle (**c**) and cell migration (**d**) were plotted

Subsequently, we further explored the potential functions of differentially expressed genes in both norm-Exo group and hypo-Exo group, we put the dysregulated mRNAs, including 839 down-regulated mRNAs and 113 up-regulated mRNAs into gene ontology (GO) biological process enrichment and Kyoto Encyclopedia of Genes and Genomes (KEGG) signaling pathways analysis. According to Fig. 7a, these genes were significantly enriched in cancer-related GO terms, such as cell proliferation (GO: 0008283), cell cycle (GO: 0007049), migration (GO: 0016477) and angiogenesis (GO: 0001525). Moreover, KEGG signaling pathways analysis showed that dysregulated mRNAs also play a role in cancer-related pathways such as cell cycle (has: 04110) and NOD-like receptor signaling pathway (hsa:04621) (Fig. 7b). Then we plotted the PPI network of dysregulated mRNAs enriched in cell cycle (GO: 0007049) (Fig. 7c) and cell migration (GO: 0016477) (Fig. 7d). Through calculating the connectivity degree of genes in the cell cycle network, we identified the hub genes PLK1, BUB1 and AURKA which were considered highly correlated with other genes in the network and played critical roles in modulating cell cycle (Fig. 7c). Similarly, VEGFA, CXCL8 and CCL2 were hub genes in in the cell migration network (Fig. 7d).

## Discussion

Hypoxia, or low oxygen tension, has emerged as a specific and general feature of the tumor microenvironment which contribute to cancer development and aggressiveness [19]. In oncology, it still remains to be elucidated that how cancer cells adapt to the hypoxia environment. Actually, hypoxic microenvironment induces a series of adaptive mechanisms including phenotypic modulation of stromal cells in the tumor microenvironment that can prolong the survival and mediate the dissemination of malignant cells [31, 32]. Importantly, hypoxia activates the angiogenic signaling pathway and results in the sprouting of blood vessels from the surrounding tissues into the tumor, during which the intercellular communication between cancer cells and endothelial cells is indispensable [33].

Exosomes are small vesicles of endocytic origin released by most cell types. Exosomes can facilitate eukaryotic intercellular communication under a wide range of normal physiological contexts. In malignancies, this regulatory circuit is co-opted to promote cancer cell survival and outgrowth [34].

Here, we provide evidence that exosomes from ESCC cells constitute a potent mediator of intercellular communication between cancer and vascular endothelial cells. We firstly characterized the exosomes from ESCC cells and visualized the internalization of these exosomes by HUVECs. Subsequently, we verified that exosomes derived from ESCC cells enhanced HUVECs proliferation through regulating its cell cycle. The invasive ability of HUVECs was also activated by exosomes from ESCC. In vitro and in vivo assay showed that exosomes from ESCC cells significantly promoted the formation of capillary-like structures of HUVECs and improved the microvessel density in transplanted gel plugs from nude mice. Based on these results, we concluded that exosomes from ESCC cells enhanced angiogenesis in TEM.

Moreover, previous studies have demonstrated that hypoxia promotes the release of exosomes by cancer cells and the secreted proteome from hypoxic carcinoma cells are closely associated with exosomes, indicating a potential role of exosomes in regulating the hypoxic response of tumor cells [35, 36]. In the present study, we compared the biological modulatory role of exosomes from ESCC cells cultured in normoxic and hypoxic condition by performing a series function experiments aforementioned. Hypoxia augmented the angiogenic effects of exosomes derived from ESCC and resulted in the enhancement of vascular formation. These results suggested that exosomes might act as a potential regulator which participate in the hypoxia-driven, phenotypic alteration of endothelial cells in TEM and, as a result, affect angiogenesis.

It has been widely accepted that angiogenesis is closely related with tumor growth metastasis [37]. Hence, we further explored the contribution of hypoxic exosomes in ESCC angiogenesis, tumor growth and metastasis. In tumor-bearing mice model, hypoxic exosomes significantly enhanced the ESCC progression by promoting the proliferation of cancer cells, vascular formation and metastasis. Based on the results we mentioned above, we supposed that hypoxia altered the content of exosome secreted by ESCC and enhanced the vascular formation by endothelial cells after internalized these exosomes in TEM. The newly-formed vascular alleviated the hypoxia condition in TEM of ESCC and in turn contribute to the tumor growth. Moreover, increased micro vessel density in TEM facilitated the escape of cancer cells into the bloodstream. Disseminated tumor cells in the blood paved the way for follow-up establishment of metastatic colonies in secondary sites.

On the other side, tumor-derived exosomes are demonstrated to be the major drivers of the pre-metastatic niche. Previous studies demonstrated that exosomes destroyed endothelial barriers and increased vascular permeability which provide an escape route for the tumor cells to enter the circulation [38]. Besides, in the target organ, a microenvironment suitable for tumor metastasis has been created by exosomes for tumor metastasis before cancer cells transfection [39, 40]. Hence, whether hypoxia take an effect on the cancer cells secreted exosomes which change the vascular permeability and result in the pre-metastatic niche formation in ESCC deserves further study.

Furthermore, exosomes carry genetic messages, such as mRNA, miRNA, lncRNA and circular RNA, and can be internalized by the recipient cells [10]. The mRNAs presented in exosomes has been demonstrated to be functional and translatable, which means they are capable of encoding polypeptides and supporting protein synthesis in host cells. Specific protein production may provide the necessary signal(s) to modulate the function of the recipient cells [41, 42]. The regulatory capacity of noncoding RNAs, such as lncRNA and miRNA, in the exosomes have also been found as extensive. Noncoding RNAs in the exosomes may act as ceRNAs and interfere with mRNAs [43]. Hence, in order to explore the mechanisms underlying the phenotypic modulation effects of exosomes in angiogenesis, we performed the comprehensive analyses of the transcriptome in HUVECs after the internalization of hypoxic and normoxic ESCC exosomes.

As we have mentioned above, hypoxia facilitate the angiogenic effects of exosomes, we focused on the RNAs which experienced an up-or down regulation in both of the two groups. We applied gene annotation and pathway enrichment analysis on the mRNAs to identify their functions. Results showed that these mRNAs were significantly enriched in biological processes such as cell proliferation and migration, and pathways such as cell cycle. Hub genes were also identified which were considered as topologically important to the structure of the network and played crucial roles in the biological process. In cell cycle term (GO: 0007049), PLK1, BUB1 and AURKA were identified as hub genes in cell cycle regulation. Previous studies demonstrate that PLK1-mediate the activation of phosphorylates glucose-6-phosphate dehydrogenase which is critical for the promoting the cell cycle progression and tumor growth in liver cancer and cervical cancer [44]. Moreover, BUB1–PLK1 complex mediate the phosphorylation of Cdc20 and inhibit the anaphase-promoting complex or cyclosome (APC/C) which result in the promotion of spindle checkpoint signaling in cervical cancer and osteosarcoma [45]. Silence of AURKA inhibits tumor growth by inducing apoptosis and G2/M cell cycle arrest in human osteosarcoma and breast cancer [46, 47].

VEGFA, CXCL8 and CCL2 were identified as the hub genes in cell migration process (GO: 0016477). It is generally accepted that VEGFA play key roles in angiogenesis [48]. The epithelial-mesenchymal transition effect of VEGFA is also verified in cancer and retinal pigment epithelial cells [49, 50]. CCL2 and CXCL8 induces epithelial-mesenchymal transition in colon cancer and bladder cancer [51, 52]. In view of this, we hypothesized that hypoxia might alter the transcriptome of exosomes from cancer cells and mediated the dysregulation of effector molecules, which triggered a series of cascade reaction, in host cells-HUVECs after internalization. Hence, we next planned to identify the hub genes which induced the angiogenesis-related signaling pathway activation and resulted in phenotype alteration of endothelial cells in the hypoxic TEM of ESCC.

## Conclusions

In summary, our data suggests that exosomes in ESCC represents a potentially targetable regulating factor in the hypoxia-driven tumor development. This study sheds light on the possible application of exosomes to fight against cancer progression and metastasis.

## Additional files

Additional file 1: Differentially expressed RNA between control group and hypo-Exo group. (XLSX 2643 kb)

Additional file 2: Differentially expressed RNA between control group and norm-Exo group. (XLSX 1939 kb)

## Data Availability

The datasets generated during the current study are available in the Additional files 1 and 2.

## Abbreviations

ESCC: Esophageal squamous cell carcinoma
FBS: Fetal bovine serum
GO: Gene ontology
HUVECs: Human umbilical vein endothelial cells
KEGG: Kyoto Encyclopedia of Genes and Genomes
LncRNA: Long non-coding RNA
PPI: Protein–protein interaction
STRING: Search Tool for the Retrieval of Interacting Genes
TEM: Tumor microenvironment
